# Exosome-Mediated eCIRP Release From Macrophages to Induce Inflammation in Sepsis

**DOI:** 10.3389/fphar.2021.791648

**Published:** 2021-12-06

**Authors:** Atsushi Murao, Chuyi Tan, Alok Jha, Ping Wang, Monowar Aziz

**Affiliations:** ^1^ Center for Immunology and Inflammation, The Feinstein Institutes for Medical Research, Manhasset, NY, United States; ^2^ Departments of Surgery and Molecular Medicine, Zucker School of Medicine at Hofstra/Northwell, Manhasset, NY, United States

**Keywords:** eCIRP, exosomes, sepsis, cytokines, macrophage, neutrophil

## Abstract

Extracellular cold-inducible RNA-binding protein (eCIRP) is an important damage-associated molecular pattern (DAMP). Despite our understanding of the potentially harmful effects of eCIRP in sepsis, how eCIRP is released from cells remains elusive. Exosomes are endosome-derived extracellular vesicles, which carry proteins, lipids, and nucleic acids to facilitate intercellular communication and several extracellular functions. We hypothesized that eCIRP is released via exosomes to induce inflammation in sepsis. Exosomes isolated from the supernatants of LPS-treated macrophage culture and serum of endotoxemia and polymicrobial sepsis mice showed high purity, as revealed by their unique median sizes ranging between 70 and 126 nm in diameter. eCIRP levels of the exosomes were significantly increased after LPS treatment in the supernatants of macrophage culture, mouse serum, and cecal ligation and puncture (CLP)-induced sepsis mouse serum. Protease protection assay demonstrated the majority of eCIRP was present on the surface of exosomes. Treatment of WT macrophages and mice with exosomes isolated from LPS-treated WT mice serum increased TNFα and IL-6 production. However, treatment with CIRP^−/-^ mice serum exosomes significantly decreased these levels compared with WT exosome-treated conditions. CIRP^−/-^ mice serum exosomes significantly decreased neutrophil migration *in vitro* compared with WT exosomes. Treatment of mice with serum exosomes isolated from CIRP^−/-^ mice significantly reduced neutrophil infiltration into the peritoneal cavity. Our data suggest that eCIRP can be released via exosomes to induce cytokine production and neutrophil migration. Thus, exosomal eCIRP could be a potential target to inhibit inflammation.

## Introduction

Cold-inducible RNA-binding protein (CIRP) is an 18-kD stress-responsive glycoprotein primarily present in the nucleus under normal conditions (Nishiyama et al., 1997). Under stressed conditions such as hypothermia, hypoxia, and ultra-violate irradiation, it migrates from the nucleus to the cytoplasm to act as an intracellular RNA chaperone (Wellmann et al., 2004; Zhu et al., 2016). We have recently identified that CIRP is also present in the extracellular space while stimulating macrophages with hypoxia and lipopolysaccharides (LPS) (Qiang et al., 2013). We further determined the presence of extracellular CIRP (eCIRP) in the blood of humans and mice with various clinical conditions such as sepsis, ischemia/reperfusion (I/R) injuries, and hemorrhagic shock (Qiang et al., 2013; Cen et al., 2017; Gurien et al., 2020). Hypoxia induces CIRP expression and release by activating the transcription factor, hypoxia-inducible factor 1α (HIF1α), while LPS induces CIRP expression and release through toll-like receptor 4 (TLR4) and nuclear factor-κB (NF-κB) pathway (Aziz et al., 2019). Unlike the release mechanism of cytokines and chemokines mediated through their N-terminal signal peptide, CIRP does not have a leader sequence to be released from cells during inflammation (Aziz et al., 2019). We previously reported that following hypoxia exposure to the macrophages, intracellular CIRP migrates from the nucleus to the cytoplasm, fuses with stress granules, and then is released *via* lysosomal secretion (Qiang et al., 2013). This finding enlightened the possibility of CIRP’s secretion through a mechanism that causes loading or connecting to the vesicles/granules to be actively released from cells.

Following its secretion from cells, eCIRP acts as an important damage-associated molecular pattern (DAMP) (Zindel and Kubes, 2020). DAMPs are endogenous molecules which activate the immune system by stimulating pattern recognition receptors (PRRs) to contribute to various diseases (Zindel and Kubes, 2020). eCIRP induces inflammation by binding to its receptors such as TLR4 and triggering receptor expressed on myeloid cells-1 (TREM-1) (Qiang et al., 2013; Denning et al., 2020). Macrophages and neutrophils treated with eCIRP release pro-inflammatory cytokines, chemokines, and extracellular traps (ETs) (Aziz et al., 2019; Ode et al., 2019; Murao et al., 2020a). Normal mice injected with eCIRP exhibit systemic inflammation and acute lung injury (ALI), while CIRP^−/−^ mice are protected from sepsis, I/R injuries, and other inflammatory diseases (Qiang et al., 2013; Denning et al., 2020; Gurien et al., 2020). Recent studies identified several antagonists of CIRP to inhibit eCIRP’s binding to its receptors, TLR4 and TREM-1 (Qiang et al., 2013; Denning et al., 2020). We found that these antagonists abrogated inflammation and tissue injury in various diseases. Thus, eCIRP plays a significant role in inflammation, and targeting eCIRP is a potential approach to mitigate inflammatory diseases.

DAMPs are released passively by cell death as well as actively via exocytosis of secretory lysosomes and exosomes (Murao et al., 2021). Exosomes are 30–150 nm extracellular vesicles (EV) with lipid bilayer membrane structures carrying proteins, lipids, and nucleic acids to facilitate intercellular communication (Murao et al., 2020b; Kalluri and Lebleu, 2020). Following endocytosis, inward invagination of endosome gives rise to the generation of intraluminal vesicles (ILVs), future exosomes, concomitant with the cargo sorting. The subset of endosomes containing ILVs is called multivesicular bodies (MVBs). Exosomes are released into the extracellular space when the MVBs fuse with the plasma membrane (Murao et al., 2020b; Kalluri and Lebleu, 2020). Released exosomes migrate to the site of action and are absorbed by the recipient cells to be eliminated from the body through metabolism and excretion (Kim et al., 2019). Almost all cell types secrete exosomes, and the exosomes are found in various biological fluids, such as blood, urine, saliva, and breast milk (de la Torre Gomez et al., 2018). Studies have shown that exosomes carry different DAMPs, including high mobility group box 1 (HMGB1), heat shock proteins, histones, adenosine triphosphate, extracellular RNA, and cell-free DNA. In fact, the release of DAMPs via exosomes can be upregulated by stimulation of the cells with LPS (Sakaki et al., 2013; Tulapurkar et al., 2015; Balusu et al., 2016; Nair et al., 2018; Li et al., 2020). Exosomal DAMPs are functionally active and contribute to the pathogenesis of inflammatory diseases by inducing cytokine production, immune cell polarization, and cell death (Murao et al., 2020b).

The current study tested our hypothesis that eCIRP is released through exosomal secretion to contribute to inflammation by inducing cytokine production by the macrophages and neutrophil migration. Thus, identifying a new mechanism of eCIRP’s release through exosomes may reveal a novel therapeutic avenue to mitigate inflammation by controlling eCIRP’s release from cells during inflammation.

## Materials and Methods

### Mice

Male 8–12-week-old wild-type (WT) C57BL/6 mice were purchased from Charles River (Charles River, Wilmington, MA) and were acclimated to the environment for 5–7 days before we performed studies with them. CIRP^−/−^ mice (C57BL/6 background) initially obtained from Jun Fujita (Kyoto University, Kyoto, Japan) were bred and maintained in our facility. Mice were housed in a temperature-controlled room on a 12-h (h) light-dark cycle and fed a standard mouse chow diet. All experiments were performed following the guidelines for using experimental animals by the National Institutes of Health and were approved by our Institutional Animal Care and Use Committees (IACUC).

### *In vivo* LPS Administration

WT and CIRP^−/−^ mice were intraperitoneally (*i.p*) injected with 5 mg/kg LPS (MilliporeSigma, Burlington, MA). After 4 h of the injection of LPS, mice were anesthetized, and blood was drawn to collect the serum for isolating exosomes.

### Mouse Model of Sepsis

Polymicrobial sepsis was induced in mice by cecal ligation and puncture (CLP) (Gurien et al., 2020). WT mice were anesthetized with isoflurane and a midline abdominal incision was created. The cecum was ligated with a 4–0 silk suture 1 cm proximal from its distal extremity and punctured twice with a 22-gauge needle. The wound was closed in layers. Sham animals underwent a laparotomy without cecal ligation and puncture. Following the surgery, 1 ml of normal saline was subcutaneously injected to avoid surgery-induced dehydration. After 20 h of the surgery, the serum was harvested to isolate exosomes.

### Cell Culture and Isolation of Primary Mouse Peritoneal Macrophages

Mouse macrophage cell line RAW 264.7 cells were obtained from ATCC. Murine peritoneal macrophages were isolated from WT mice (Denning et al., 2020). Briefly, mice were euthanized using CO_2_ asphyxiation, and peritoneal cells were collected using peritoneal lavage with PBS. Total peritoneal cells were isolated by centrifugation at 200 *g* for 10 min and subsequently cultured in DMEM (Thermo Fisher Scientific, Waltham, MA). After 4 h nonadherent cells were removed, and adherent cells, primarily macrophages, were collected, counted, and plated in the cell culture plate for subsequent studies.

### Isolation of Exosomes

Exosomes were isolated from cell culture media and serum using a total exosome isolation kit (Themo Fisher Scientific) by following the manufacturer’s protocol. Isolated exosomes were resuspended in PBS. The protein concentrations of the exosomes were determined by the Bio-Rad protein assay reagent (Hercules, CA). The size distribution of the exosomes was assessed by nanoparticle tracking analysis (NTA) using ZetaView TWIN (Particle Matrix, Inning am Ammersee, Germany), and the histogram was created with FlowJo software (Tree Star, Ashland, OR). NTA determines the size of nanoparticles within the range of 10–1,000 nm by using a special algorithm based on their movement recorded by a light sensitive camera under a laser illumination.

### Assessment of Exosomal Levels of eCIRP *in vitro*

RAW 264.7 cells were cultured in DMEM (Thermo Fisher Scientific) and stimulated with 1 μg/ml LPS for 20 h. Exosomes were isolated from the culture supernatants, and the protein amount of the exosomes was determined by a Bio-Rad protein assay reagent. Equal protein amounts of the exosomes were supplemented with Lane Marker Non-Reducing Sample Buffer (Thermo Fisher Scientific), which contains SDS sufficient to lyse exosomes to release and denature the proteins. The crude exosomal proteins contained in the sample buffer were then subjected to Western blotting. The blots were reacted with anti-CIRP Ab (Proteintech, Rosemont, IL) followed by fluorescent-labeled secondary Abs (Li-Cor Biosciences, Lincoln, NE), and detection was done using an Odyssey FC Dual-Mode Imaging system (Li-Cor Biosciences, Lincoln, NE). The densitometry intensities of the bands were measured using ImageJ software (NIH). The blots were also reacted with anti-CD63 Ab (Abcam, Cambridge, UK) followed by the same procedures for the loading control.

### Assessment of Exosomal Levels of eCIRP *in vivo*

Exosomes isolated from the serum of LPS-injected or CLP mice were lysed using extraction buffer containing 25 mM Tris, 0.15 M NaCl, 1 mM EDTA, 1% NP-40, 5% glycerol, 2 mM Na_3_VO_4_, and protease inhibitor cocktail (Roche Diagnostics, Basel, Switzerland), at pH 7.4. Protein concentration was determined by the Bio-Rad protein assay reagent. CIRP levels of exosomal lysates were assessed using mouse CIRP ELISA Kit (LSBio, Seattle, WA) and normalized by protein concentrations.

### Assessment of the Effect of Exosome Inhibitor GW4869 on the Release of eCIRP

RAW 264.7 cells were cultured in Opti-MEM (Thermo Fisher Scientific) and pre-treated with GW4869 (MilliporeSigma) at a dose of 10 μg/ml for 2 h followed by 20 h stimulation with 1 μg/ml LPS. An equal volume of the supernatants was supplemented with Lane Marker Non-Reducing Sample Buffer (Thermo Fisher Scientific) to perform Western blotting for CIRP. LPS at a dose of 5 mg/kg was intraperitoneally injected to WT mice with or without 1 mg/kg GW4869 injection simultaneously. Serum was harvested 4 h after the injection and eCIRP levels of the serum were assessed by mouse CIRP ELISA Kit (LSBio). CLP was performed in WT mice with or without intraperitoneal instillation of GW4869 before closing the abdomen. 20 h after CLP, serum was harvested to assess eCIRP levels by mouse CIRP ELISA Kit (LSBio).

### Homology Modeling of CIRP and CD63

The amino acid sequences of mouse CIRP (P60824) and CD63 (P41731) were retrieved from the Uniprot database. The models were generated using Iterative Threading ASSEmbly Refinement (I-TASSER) server based on templates identified by threading approach to maximize the percentage identity, sequence coverage, and confidence (Yang et al., 2015). The CIRP structure has RNA binding domain (aa 6–84), disordered region (aa 70–172), and polar residues (aa 143–172). The CD63 has different topological and transmembrane domains and a lysosomal targeting motif (234–238). Models were refined based on repetitive relaxations by short molecular dynamics simulations for mild (0.6 ps) and aggressive (0.8 ps) relaxations with 4 fs time step after structure perturbations. The model refinement enhanced specific parameters, including Rama favored residues and a decrease in poor rotamers.

### Protein-Protein Docking

The docking of CIRP and CD63 protein structure models was performed using ATTRACT tool (Schindler et al., 2015), which uses an approach of conformational flexibility of binding partners. The docking process includes pre-calculation of potential energy on a grid and then interactions are calculated by interpolation from nearest grid points. Moreover, docking process includes several Monte Carlo simulations or energy minimization steps.

### Protein-Protein Interaction

The analysis of CIRP-CD63 complex interactions were calculated using PDBePISA tool (Krissinel and Henrick, 2007). The surface area of interaction interface and thermodynamic parameters were calculated. The complex structure was visualized using PyMOL and Chimera (Sanner et al., 1996).

### Assessment of Intracellular Interaction Between CIRP and CD63

RAW 264.7 cells (1 × 10^6^ cells) were stimulated with 1 μg/ml LPS for 20 h. We also isolated cells from the peritoneal lavage of mice 4 h after LPS injection and 20 h after CLP. The cells were lysed using a buffer containing 25 mM Tris, 0.15 M NaCl, 1 mM EDTA, 1% NP-40, 5% glycerol, 2 mM Na_3_VO_4_, and protease inhibitor cocktail (Roche Diagnostics), pH 7.4. The extracted proteins were immunoprecipitated with anti-CD63 Ab (Abcam) and protein A/G plus agarose beads (Themo Fisher Scientific), and the eluted samples were subjected to Western blotting. Finally, the blots were detected with anti-CIRP Ab (Proteintech). Total lysates were subjected to Western blotting, and the blots were detected with anti-CD63 Ab (Abcam) for internal control.

### Protease Protection Assay

Equal amounts of exosomes isolated from the supernatants of LPS-treated RAW 264.7 cells were separated into 3 groups: 1. control, 2. 100 ng/ml protease K (Thermo Fisher Scientific), 3. 1% Triton-X 100 + 100 ng/ml protease K. The samples were incubated in 37°C for 30 min and denatured with 99°C Lane Marker Non-Reducing Sample Buffer (Themo Fisher Scientific) for Western blotting. The blots were reacted with anti-CIRP Ab (Proteintech) and anti-TSG101 Ab (Cell Signaling Technology, Danvers, MA). TSG101 is an important component of the endosomal sorting complex required for transport (ESCRT) machinery and serves as an internal control of exosomes (Fan et al., 2020).

### Culture of Macrophages With WT and CIRP^−/−^ Exosomes

RAW 264.7 cells and peritoneal macrophages were co-cultured with 5 μg/ml of exosomes isolated from WT or CIRP^−/−^ mice treated with PBS or LPS. After 20 h supernatants were analyzed by ELISA kits specific for IL-6 and TNFα (both from BD Biosciences, Franklin Lakes, NJ) according to the manufacturer’s protocol.

### Intraperitoneal Injection of WT vs CIRP^−/−^ Exosomes in Mice

WT mice were intraperitoneally injected with 10 mg/kg of exosomes isolated from WT or CIRP^−/−^ mice challenged with LPS. After 4 h of injection with exosomes, serum and peritoneal lavage were collected. Serum was analyzed by ELISA for IL-6 and TNFα (both from BD Biosciences). The number of peritoneal neutrophils labeled with anti-Ly6G Ab (BioLegend, San Diego, CA) was counted by FACSymphony Flow Cytometer (BD Biosciences) using Precision Count Beads (BioLegend).

### Neutrophil Migration Assay With WT vs CIRP^−/−^ Exosomes

Neutrophils were isolated from the bone marrow of WT mice using the EasySep mouse neutrophil enrichment kit (STEMCELL, Vancouver, BC). Neutrophils (0.5 ×10^6^) were added into the 3.0 μm cell culture insert (Corning, Corning, NY) placed on the cell culture plate. Exosomes were isolated from WT and CIRP^−/−^ mice challenged with LPS, and exosomal protein concentration was determined. Exosomes (5 μg/ml) were added into the bottom chamber of the 24-well plate containing insert loaded with neutrophils. After 4 h, the number of migrated neutrophils in the well was counted by flow cytometry using anti-Ly6G Ab (BioLegend) and Precision Count Beads (BioLegend) as described earlier.

### Statistical Analysis

Data represented in the figures are expressed as mean ± SEM. ANOVA was used for one-way comparison among multiple groups, and the significance was determined by the Student Newman-Keuls (SNK) test. The paired Student t-test was applied for two-group comparisons. Significance was considered for *p* ≤ 0.05 between study groups. Data analyses were carried out using GraphPad Prism graphing and statistical software (GraphPad Software, San Diego, CA).

## Results

### Endotoxin and Sepsis Increase eCIRP Release *via* Exosomes

**FIGURE 1:**
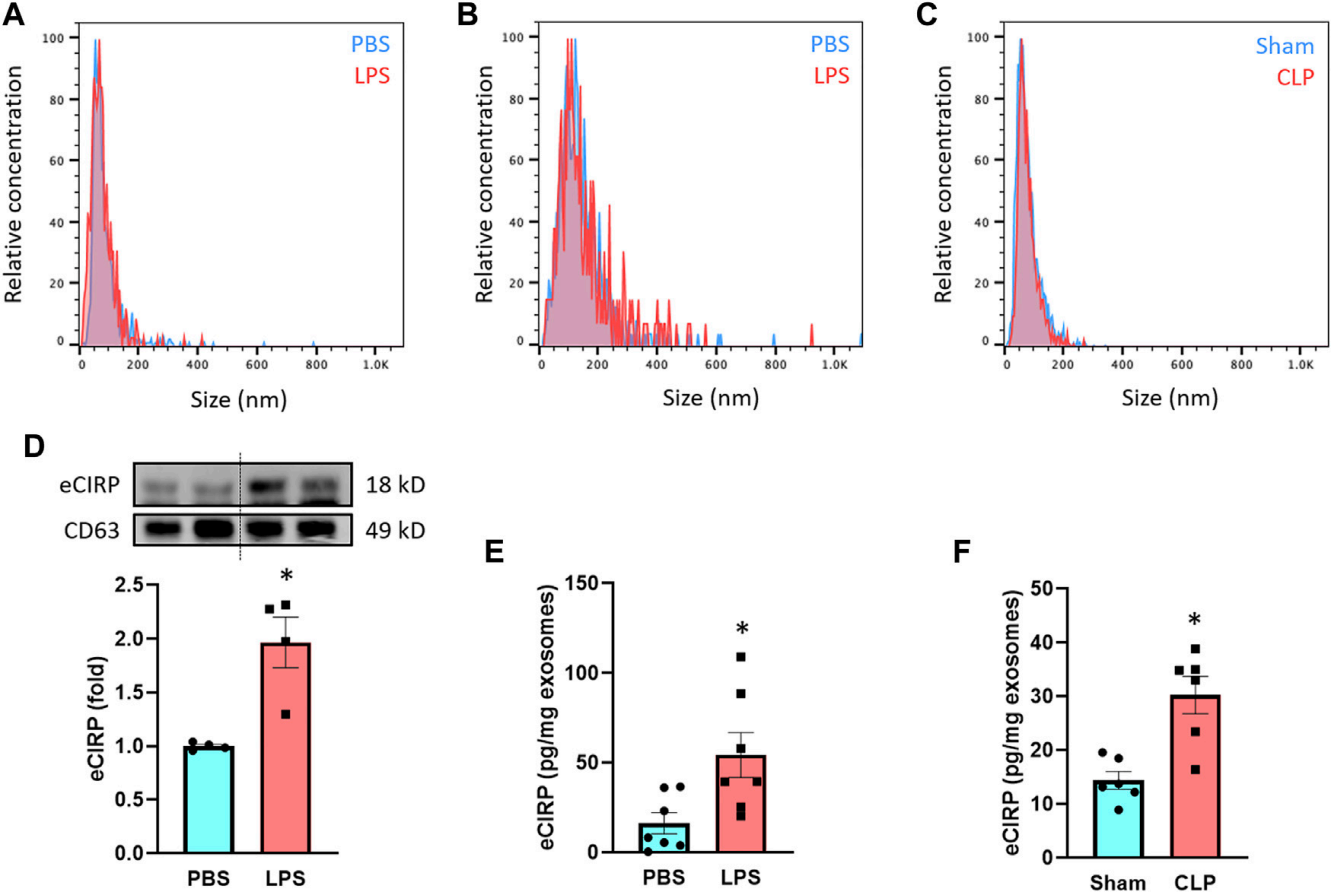
LPS induces eCIRP release via exosomes. The size distribution of the isolated exosomes from **(A)** culture supernatants of PBS/LPS-treated RAW 264.7 cells and serum of **(B)** PBS/LPS-injected or **(C)** Sham/CLP mice was assessed by ZetaView TWIN. The experiments were repeated three times. **(D)** eCIRP levels of the exosomes isolated from the culture supernatants of PBS- or LPS-treated RAW 264.7 cells were determined by Western blotting. RAW 264.7 cells (10^6^) were treated with PBS or LPS (1 µg/ml). After 20 h of LPS stimulation, exosomes were isolated from culture supernatants. Protein contents were determined in the exosomes, and an equal amount of protein (30 µg) was loaded in the gel to assess eCIRP levels by Western blot using anti-CIRP Ab. Blots were stripped off and re-probed with anti-CD63 Ab to serve as a loading control. Data are expressed as means ± SE (*n* = 4 samples/group). The groups were compared by paired student’s t-test (*p < 0.05 vs. PBS). **(E)** eCIRP levels of the exosomes isolated from the serum of PBS- or LPS-injected mice were determined by ELISA and were normalized with the protein concentration. Data are expressed as means ± SE (*n* = 7 samples/group). The groups were compared by paired student’s t-test (*p < 0.05 vs. PBS). **(F)** eCIRP levels of the exosomes isolated from the serum of sham or CLP mice were determined by ELISA and were normalized with the protein concentration. Data are expressed as means ± SE (*n* = 6 samples/group). The groups were compared by paired student’s t-test (*p < 0.05 vs. Sham).

We treated macrophages and mice with LPS and then collected exosomes from culture supernatants and serum to assess eCIRP levels within the exosomes. We first examined the quality of exosomes isolation by NTA based on their unique size range demarcating from other extracellular vesicles. The median size of the isolated exosomes was 126 and 72 nm for macrophage culture supernatant- and serum-derived samples, respectively, indicating efficient isolation and purity of the exosomes (Figures 1A,B). The size difference of the exosomes between the sources was in line with previous studies showing exosomes isolated from cell lines, including RAW 264.7 cells, exhibited larger size than primary cell- or serum-derived exosomes (Singh et al., 2012; Cai et al., 2018; Zhao et al., 2020). We also subjected mice to CLP, a model of polymicrobial sepsis. The median size of exosomes isolated from the serum of CLP mice was 70 nm, which was identical to that of LPS-injected mice (Figure 1C). There were no significant differences in the size of exosomes between PBS/Sham and LPS/CLP groups (Figures 1A–C). Next, we compared the levels of eCIRP in the exosomes released from RAW 264.7 cells subjected to PBS or LPS stimulation. While we observed basal levels of exosomal eCIRP in the PBS group, LPS stimulation significantly increased the levels of eCIRP in the exosomes (Figure 1D). We also assessed the levels of CD63, one of the pan markers of exosomes, and found that it was homogeneously present among the samples (Figure 1D). We then checked exosomal eCIRP release in mice injected with LPS. Exosomes isolated from the serum of LPS-injected mice contained significantly higher amounts of eCIRP than that of the PBS-injected mice (Figure 1E). Similarly, exosomes isolated from CLP mice serum exhibited significantly elevated levels of eCIRP compared to sham mice (Figure 1F). These findings indicate that eCIRP is released via exosomes from macrophages, and the levels of exosomal eCIRP are increased by LPS treatment of the macrophages and mice and in septic mice.

### CIRP Interacts With CD63, a Member of the Tetraspanin Family Intracellularly

**FIGURE 2:**
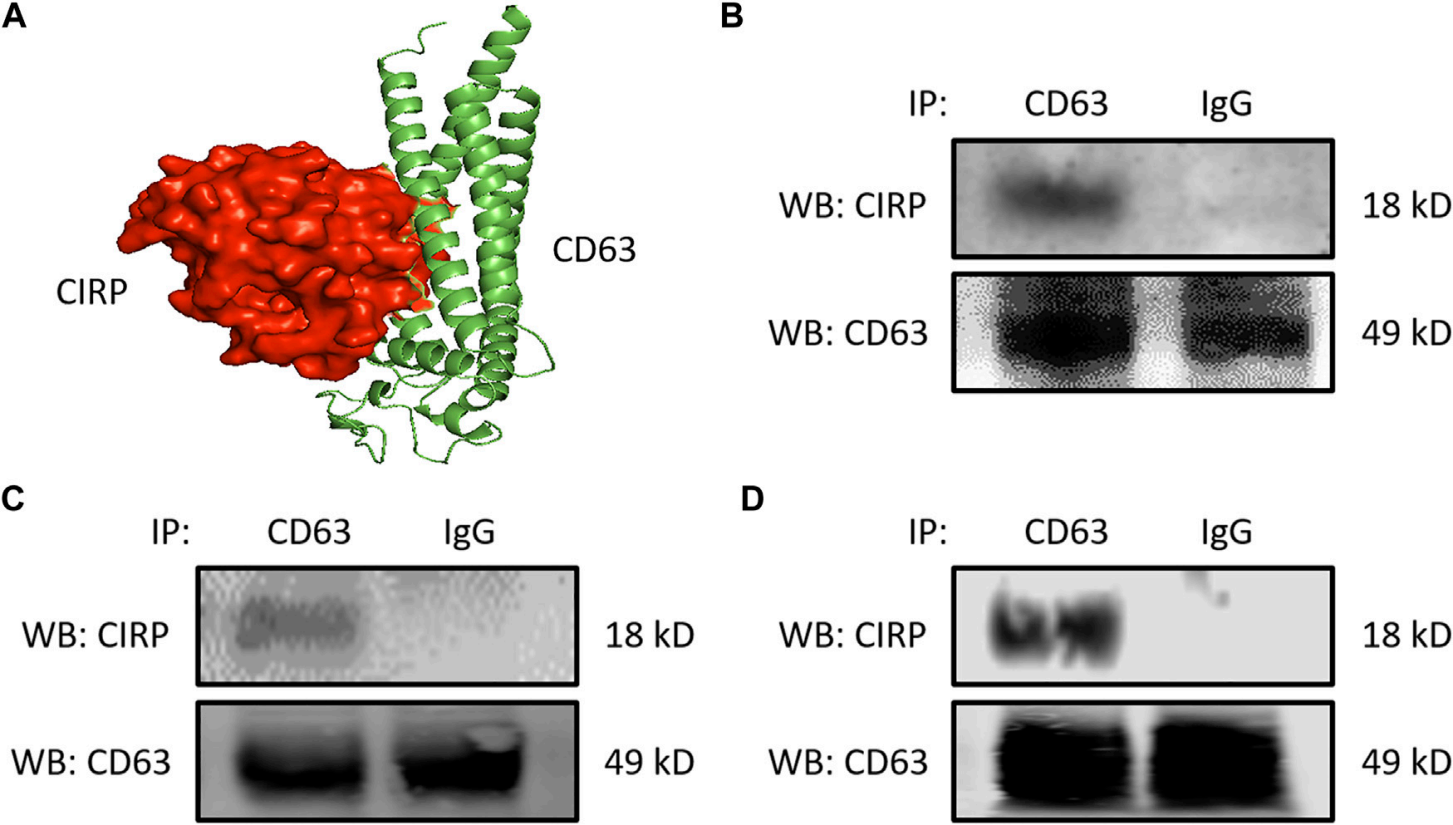
CIRP interacts with CD63, a member of the tetraspanin family intracellularly. **(A)** Molecular binding between CIRP and CD63 was predicted by 3D computational modeling. **(B)** RAW 264.7 cells challenged with LPS for 20 h and peritoneal cells isolated from mice **(C)** 4 h after LPS injection or **(D)** 20 h after CLP were lysed and immunoprecipitated with anti-CD63 Ab or IgG control. Precipitated proteins were subjected to WB of anti-CIRP Ab. CD63 levels of the total lysates were assessed by WB for the internal control. The experiment was repeated three times.

We next focused on the intracellular formation of exosomes containing CIRP. CD63 is one of the tetraspanin proteins and is enriched in the exosomes. CD63 is not only used as a biomarker of exosomes but is also involved in exosome cargo loading (Wei et al., 2021). First, we assessed the interaction between CIRP and CD63 by computational modeling, and it indeed predicted the direct binding (Figure 2A). The CIRP-CD63 structure complex showed that the disordered region and some polar residues of CIRP interacted with the 1st, 3rd, and 4th alpha-helices of the transmembrane region of CD63. The CIRP-CD63 complex structure revealed that based on the interaction interface surface area in Ȧ2, which was 1,299 Ȧ2 and other thermodynamic parameters such as free energy of binding upon complex formation (∆iG) −15.6 kcal/mol indicated that the CIRP and CD63 interactions could be an intermediate type of interactions. Moreover, the free energy of dissociation (∆Gdisso) was 3.5 kcal/mol. The positive free energy of dissociation indicated that the CIRP-CD63 complex was stable. The entropy change after dissociation (T∆S) was 12.5 kcal/mol. There was one hydrogen bond formation (Gly27-Arg75), which possibly contributed to the stability of CIRP-CD63 complex structure (Table 1). Next, we assessed their interaction in cells by immunoprecipitation. We collected the proteins of RAW 264.7 cells challenged with LPS and peritoneal cells isolated from LPS-injected or CLP mice and subjected the total proteins to immunoprecipitation of anti-CD63 Ab. We found that cell lysates immunoprecipitated with anti-CD63 Ab revealed the presence of CIRP. In contrast, the lysates immunoprecipitated with IgG did not show the existence of CIRP, indicating the intracellular interaction between CIRP and CD63 (Figures 2B–D). These results suggest the formation of the CIRP-CD63 complex and the sorting of CIRP to exosomes inside the cells.

**TABLE 1:** Interaction between CIRP and CD63 as revealed by computational analysis. The analysis of CIRP-CD63 complex interactions were calculated using PDBePISA tool. The surface area of interaction interface and thermodynamic parameters were determined.

Complex	Surface area $\left({\dot{A}}^{2}\right)$	Binding (GΔ) energy (Kcal/mol)	Free energy of dissociation $\left(\Delta G^{\mathrm{diss}}\right)$ in kcal/mol	Entropy change at dissociation $\left(T\Delta S^{\mathrm{diss}}\right)$	N_HB_	N_SB_
CIRP-CD63	1,299.0	−15.6	3.5	12.5	1	0

### An Exosome Inhibitor GW4869 Attenuates eCIRP Release

**FIGURE 3:**
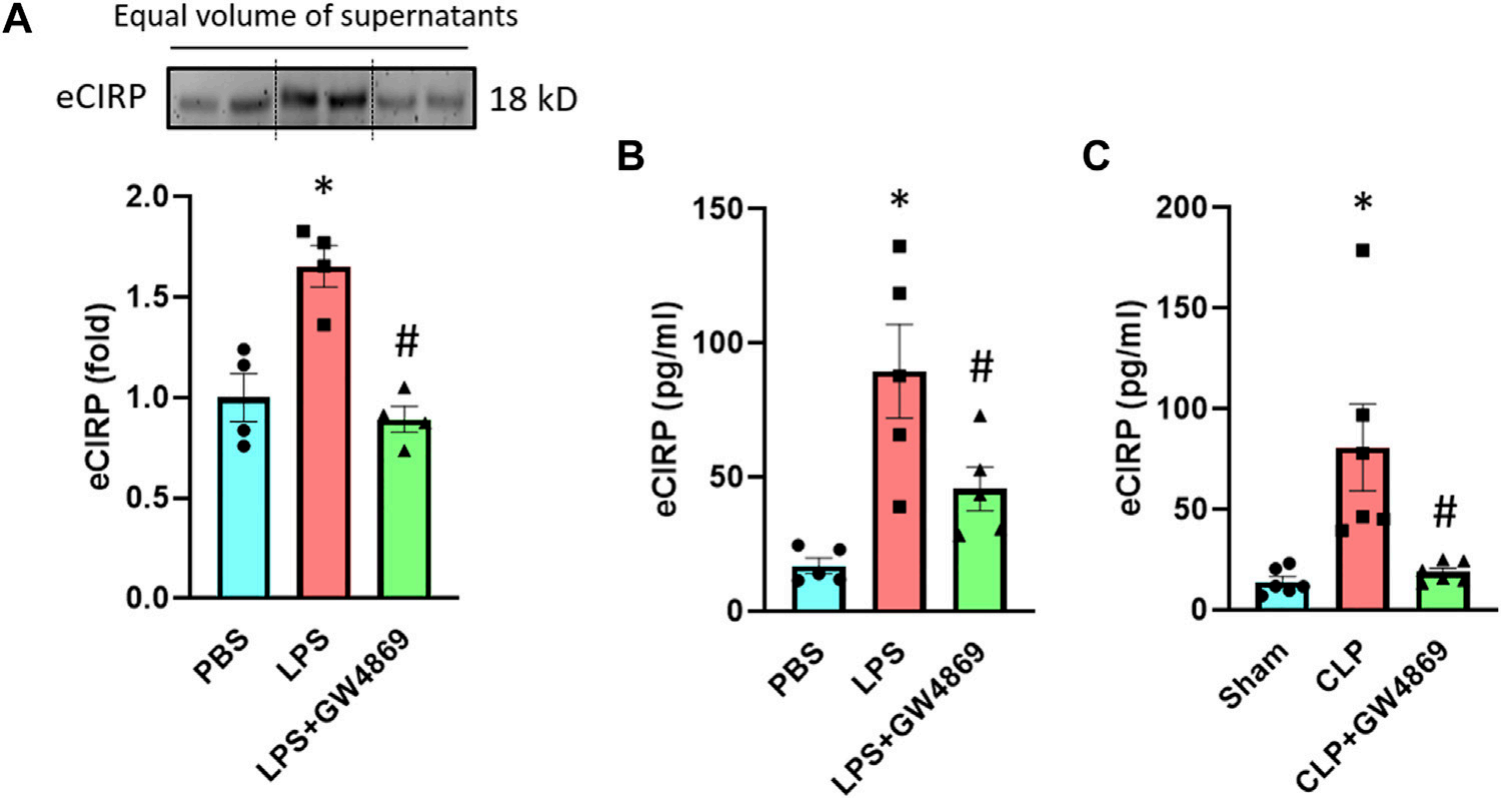
An exosome inhibitor GW4869 attenuates eCIRP release. **(A)** RAW 264.7 cells were treated with PBS, LPS or LPS + GW4869. After 20 h eCIRP levels of the supernatants were assessed by Western blotting. Data are expressed as means ± SE (*n* = 4 samples/group). The groups were compared by two-way ANOVA and SNK method (^*^ *p* < 0.05 vs PBS; ^#^ *p* < 0.05 vs LPS). **(B)** Mice were injected with PBS, LPS or LPS + GW4869. After 4 h the serum was harvested and eCIRP levels were assessed by ELISA. Data are expressed as means ± SE (*n* = 5 samples/group). The groups were compared by two-way ANOVA and SNK method (^*^ *p* < 0.05 vs PBS; ^#^ *p* < 0.05 vs LPS). **(C)** Mice were subjected to sham surgery, CLP or CLP with GW4869 instillation. After 20 h the serum was harvested and eCIRP levels were assessed by ELISA. Data are expressed as means ± SE (*n* = 6 samples/group). The groups were compared by two-way ANOVA and SNK method (^*^ *p* < 0.05 vs Sham; ^#^ *p* < 0.05 vs CLP).

To further confirm eCIRP’s release *via* exosomes, we used GW4869, an inhibitor of exosome biogenesis and release (Catalano and O’driscoll, 2020). We pre-treated the RAW 264.7 cells with GW4869 for 2 h and stimulated these cells with LPS for 20 h. Pre-treatment with GW4869 significantly decreased the levels of eCIRP in the culture supernatants of macrophages stimulated with LPS (Figure 3A). We also assessed the effect of GW4869 on eCIRP release in endotoxemic and septic mice. While LPS increased eCIRP levels in mice serum, GW4869 injection significantly attenuated the levels of eCIRP in the serum of LPS-injected mice (Figure 3B). eCIRP was significantly elevated in the serum of CLP-induced septic mice, whereas GW4869 dramatically decreased eCIRP levels in the serum of CLP mice (Figure 3C). Collectively, this data, along with the data of intracellular interaction between CIRP and CD63 (Figure 2), confirmed exosomes to be the means of eCIRP release instead of the possibility that exosomes and eCIRP make complex in the extracellular space following their release from cells separately. In addition, the significance of the decrease of eCIRP levels back to the baseline by GW4869 treatment indicated that exosomes could play a significant role in releasing the majority of eCIRP.

### CIRP is Present on the Surface of Exosomes

**FIGURE 4:**
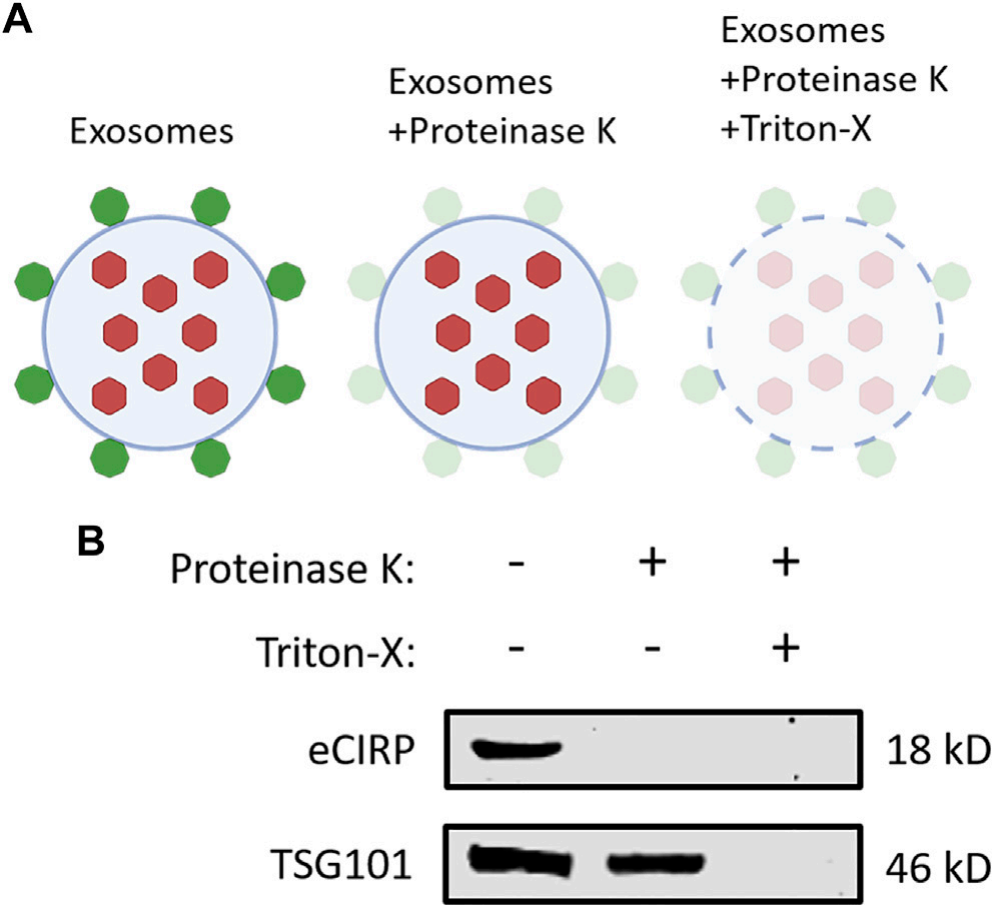
CIRP is present on the surface of exosomes. **(A)** A schematic diagram of proteinase protection assay. Proteinase K alone only digests the proteins on the surface of exosomes. An additional treatment of Triton-X with proteinase K quenches the proteins both on the surface and inside the exosomes. **(B)** Exosomes isolated from the supernatants of LPS-treated RAW 264.7 cells were incubated with Proteinase K ± Triton-X at 37°C for 30 min and subjected to Western blotting of anti-CIRP Ab and anti-TSG101 Ab. The experiment was repeated three times.

To elucidate eCIRP’s localization on exosomes, we performed a protease protection assay (Figure 4A). Exosomes isolated from the supernatants of LPS-challenged RAW 264.7 cells were treated with or without proteinase K and Triton-X 100. Proteinase K alone quenched CIRP even the membrane integrity of the exosomes was preserved in the absence of Triton-X 100. On the contrary, TSG101, one of the intra-exosomal proteins, required Triton-X 100 to be digested by proteinase K (Figure 4B). These results indicate that eCIRP is present on the surface of the exosomes, suggesting it can directly interact with cell surface receptors of eCIRP to promote eCIRP-mediated inflammation.

### Exosomal eCIRP Induces Pro-inflammatory Cytokine Production

**FIGURE 5:**
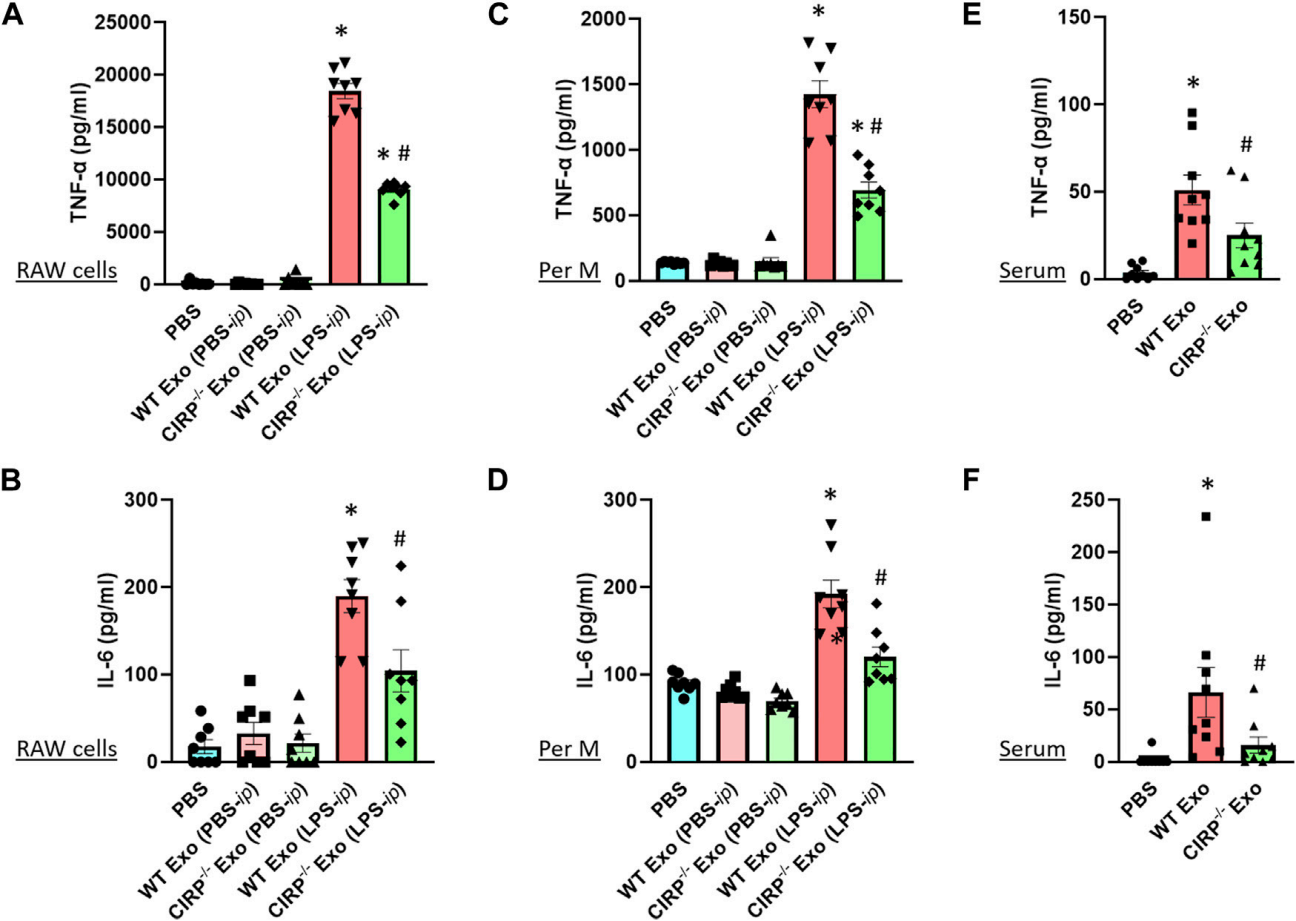
Exosomal eCIRP induces pro-inflammatory cytokine production. **(A,B)** RAW 264.7 cells (10^5^) and **(C,D)** peritoneal macrophages (10^5^) were co-cultured with 5 μg/ml of exosomes isolated from WT or CIRP^−/−^ mice treated with PBS or LPS. After 20 h supernatants were collected and IL-6 and TNF-α levels were assessed by ELISA. Data are expressed as means ± SE (*n* = 8 samples/group). The groups were compared by two-way ANOVA and SNK method (^*^ *p* < 0.05 vs PBS; ^#^ *p* < 0.05 vs exosomes from LPS- *i. p.* WT mice). **(E,F)** WT mice were intraperitoneally injected with exosomes isolated from WT or CIRP^−/−^ mice challenged with LPS. After 4 h serum was harvested and IL-6 and TNF-α levels were assessed by ELISA. The groups were compared by two-way ANOVA and SNK method Data are expressed as means ± SE (*n* = 9 samples/group). The groups were compared by two-way ANOVA and SNK method (^*^ *p* < 0.05 vs PBS; ^#^ *p* < 0.05 vs. WT exosomes).

Previous studies showed that exosomes isolated from LPS-treated macrophages or mice exhibited inflammatory responses when treated to the naive immune cells (McDonald et al., 2014). To determine the impact of exosomal eCIRP on macrophages, we first isolated exosomes from the blood of LPS-treated WT or CIRP^−/-^ mice. Those exosomes contained barely detectible endotoxin contamination (data not shown), which was consistent with a previous study (Li et al., 2018). We then treated RAW 264.7 cells separately with the WT or CIRP^−/-^ mice exosomes for 20 h and assessed the levels of cytokines in the culture supernatants. We found that exosomes isolated from PBS-injected WT mice did not induce cytokine production when added to the macrophages. On the other hand, exosomes isolated from LPS-injected WT mice induced IL-6 and TNFα production by the macrophages (Figures 5A,B). Interestingly, RAW 264.7 cells stimulated with exosomes isolated from LPS-treated CIRP^−/−^ mice blood significantly decreased IL-6 and TNFα production compared to WT exosomes (Figures 5A,B). Similarly, primary mouse peritoneal macrophages treated with exosomes obtained from LPS-injected CIRP^−/−^ mice blood showed a significant decrease in IL-6 and TNFα levels in the culture supernatants compared to exosomes isolated from LPS-injected WT mice blood (Figures 5C,D). These data indicate that eCIRP present in the exosomes induces inflammation in macrophages. To determine the role of eCIRP on exosome-mediated inflammation in mice, we *i. p.* administered WT mice with the exosomes isolated from LPS-treated WT or CIRP^−/−^ mice blood. We found that normal mice injected (*i.p.*) with exosomes isolated from LPS-treated WT mice exhibited higher levels of IL-6 and TNFα in the serum than mice injected (*i.p.*) with exosomes isolated from LPS-treated CIRP^−/−^ mice blood (Figures 5E,F). These data indicate that exosomal eCIRP induces cytokine production in mice.

### Exosomal eCIRP Promotes Neutrophil Migration

**FIGURE 6:**
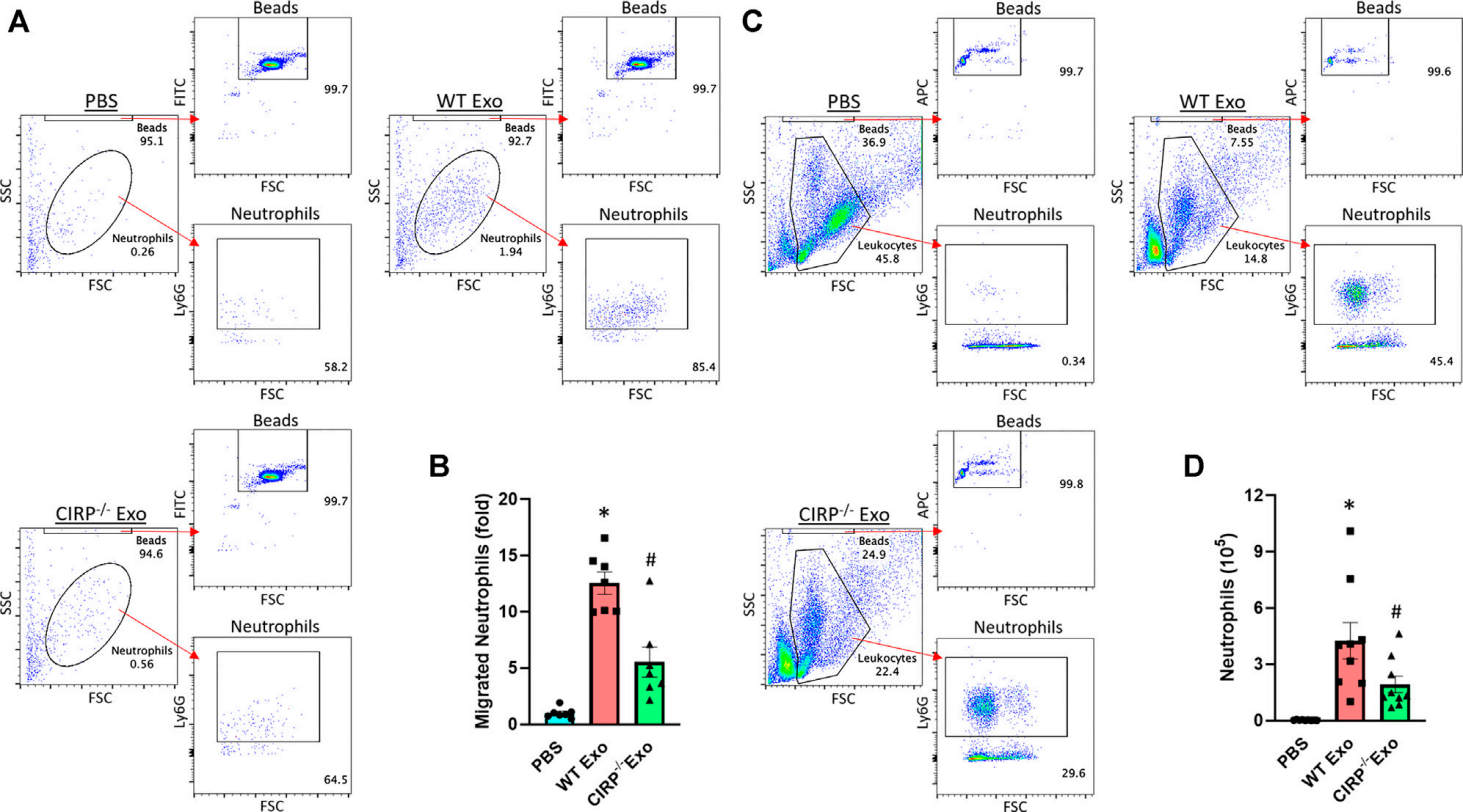
Exosomal eCIRP promotes neutrophil migration. Migration of neutrophils (5 × 10^5^) was assessed using 5 μg/ml of exosomes isolated from WT or CIRP^−/−^ mice challenged with LPS as attractants. After 4 h the number of migrated neutrophils was counted by flow cytometry and was normalized to PBS group. **(A)** Representative dot pots and **(B)** bar diagram are shown. Data are expressed as means ± SE (*n* = 7 samples/group). The groups were compared by two-way ANOVA and SNK method (^*^ *p* < 0.05 vs PBS; ^#^ *p* < 0.05 vs WT exosomes). WT mice were intraperitoneally injected with exosomes isolated from WT or CIRP^−/−^ mice challenged with LPS. After 4 h the number of infiltrated neutrophils in the peritoneal lavage was assessed by flow cytometry. **(C)** Representative dot pots and **(D)** bar diagram are shown. Data are expressed as means ± SE (*n* = 9 samples/group). The groups were compared by two-way ANOVA and SNK method (^*^ *p* < 0.05 vs PBS; ^#^ *p* < 0.05 vs WT exosomes).

We assessed the chemotactic activity of eCIRP containing exosomes. *In vitro* neutrophil migration data revealed that exosomes isolated from LPS-treated WT mice induced neutrophil migration. In contrast, exosomes isolated from LPS-treated CIRP^−/−^ mice significantly decreased the numbers of migrated neutrophils (Figures 6A,B). Furthermore, *in vivo* *i. p.* injection of exosomes isolated from LPS-treated WT mice blood increased neutrophil infiltration in the peritoneal cavity. By contrast, *i. p.* injection of exosomes of LPS-treated CIRP^−/−^ mice blood showed a significant decrease in the infiltration of neutrophils in the peritoneal cavity compared to WT exosomes treated mice (Figures 6C,D). These data indicate the direct chemotaxis effects of exosomal eCIRP.

## Discussion

The present study reveales that eCIRP is released via exosomes and has impacts on exosome-induced inflammation. Our findings on the new mechanism of eCIRP release through exosomes are crucial in inducing innate immune response since little has been known how eCIRP is released into the extracellular space, especially in terms of active secretion. As a leaderless (devoid of signal peptide sequence) protein, eCIRP is unlikely to be released via the endoplasmic reticulum (ER)-Golgi pathway (Aziz et al., 2019). Until now, eCIRP has been regarded to be released passively by necrosis or actively by secretory lysosomes (Qiang et al., 2013; Aziz et al., 2019). We have previously shown enrichment of CIRP in lysosomes in the cells under stress conditions suggesting its release by secretory lysosomes (Qiang et al., 2013). In cells, exosomes have been known to interact with lysosomes, especially for their degradation (Kalluri and Lebleu, 2020). Together with our previous and current findings, the interaction between exosomes and lysosomes in the cells might also be a process for the release of exosomal eCIRP. We also found that eCIRP is present on the surface of exosomes, suggesting its direct interaction with cell surface receptors such as TLR4 and TREM-1 to induce inflammatory responses. It was previously reported that histones present on the surface of exosomes interact with TLR4 to induce cytokine production (Nair et al., 2018). As both being nuclear proteins, eCIRP might share similar loading mechanisms with histones to be presented on the surface of exosomes. In this study, we focused on macrophages as the source of exosomal eCIRP since macrophages significantly contribute to innate immunity and have already been shown to release eCIRP under stress such as hypoxia and LPS stimulation (Qiang et al., 2013). Although the *in vitro* studies made it feasible to know the origin/source of exosomes as we studied on macrophages, in the context of *in vivo* studies, identification of the sources of exosomes that contain eCIRP is critical as any cells can release exosomes upon different kinds of stimuli (Murao et al., 2020b; Kalluri and Lebleu, 2020). Since all cell types ubiquitously express CIRP (Zhong and Huang, 2017), it is thought that under *in vivo* conditions, exosomal eCIRP can be released by various immune and non-immune cells which respond to PAMPs, DAMPs, and other stimuli. Future studies on detecting exosomal eCIRP using different stimuli on various cell types *in vitro* or using cell type-specific knock-out animals will be of interest.

Besides confirming the exosomal release of eCIRP, we also assessed the impacts of eCIRP on exosome-induced inflammation. Exosomes have already been shown to play a crucial role in sepsis because of their inflammatory function. Exosome inhibitor GW4869 attenuated IL-6 and TNFα levels in the supernatants of LPS-challenged macrophages and the serum of CLP mice (Essandoh et al., 2015). GW4869 also ameliorated sepsis-induced cardiac dysfunction and improved the survival of septic mice, indicating its clinical potential (Essandoh et al., 2015). However, the study had not revealed which exosomal contents are responsible for sepsis pathophysiology. We have found that GW4869 significantly attenuated eCIRP release in LPS-treated macrophages as well as endotoxemic and septic mice, indicating the therapeutic effects of GW4869 on sepsis could be, at least in part, due to the reduction of exosomal eCIRP. To confirm the impacts of exosomal eCIRP more directly, we have demonstrated that exosomes isolated from LPS-challenged CIRP^−/−^ mice have significantly decreased levels of inflammatory functions as compared to those of WT mice in terms of cytokine production and neutrophil migration *in vitro* and *in vivo*. It has been shown that LPS stimulation increases exosomal contents, including cytokines and DAMPs such as HMGB1, HSP, histones, ATP, and exRNAs (Sakaki et al., 2013; McDonald et al., 2014; Tulapurkar et al., 2015; Balusu et al., 2016; Nair et al., 2018; Li et al., 2020). The presence of those inflammatory molecules within the exosomes was likely to be the reason that even though the exosomes of CIRP^−/−^ mice were free from CIRP, the presence of other molecules within exosomes might have induced the inflammation, which resulted in not complete inhibition of inflammatory responses in CIRP^−/−^ exosome-treated group.

Neutrophils are the first line of defense against pathogen invasion, but their excessive activities including tissue migration cause organ injuries (Rittirsch et al., 2008). The present study has revealed that exosomal eCIRP plays a significant role in neutrophil migration. A previous study has shown that exosomes attracted neutrophils in an LTB4 receptor-dependent manner *in vitro* (Majumdar et al., 2021), indicating their direct effect on neutrophil migration, which is in line with our current finding. Furthermore, another study has shown that intraperitoneal injection of septic exosomes induced neutrophil migration in a MyD88-dependent manner (Xu et al., 2018), suggesting exosomal eCIRP stimulates TLR4/MyD88 pathway to cause neutrophil migration since eCIRP is one of the most potent ligands for TLR4. Besides the direct effect, the migration of neutrophils *in vivo* might have been amplified by chemokines released from the resident macrophages activated by the exosomes. Since exosomal eCIRP exhibited its effects on neutrophils, this finding further implicates its role in the production of reactive oxygen species (ROS), myeloperoxidase (MPO), and neutrophil extracellular traps (NETs) by neutrophils to exaggerate inflammation and tissue injury. We have previously shown that eCIRP induces the formation of NETs via TREM-1 (Murao et al., 2020a). Thus, it is likely that exosomal eCIRP also promotes NETosis in the same manner, but further studies are awaited to prove this theory.

eCIRP has been reported to be elevated in the circulation and correlate with the severity of patients suffering from sepsis (Zhou et al., 2015). Our study using exosome inhibitor GW4869 revealed that exosomal release is the major, if not all, source of eCIRP. Therefore, it is suggested that exosomal eCIRP plays a critical role in the development of inflammatory diseases. Exosomes, in general, have already shown their clinical potential. Exosomes are regarded as important biomarkers for cancer (Lebleu and Kalluri, 2020). Artificial liposomes, which share similar structures with exosomes, have already been used to deliver drugs and vaccines such as Amphotericin B and COVID-19 vaccine, respectively (Tenchov et al., 2021). Our study suggests targeting specific contents in biological exosomes, including exosomal eCIRP can be a novel therapeutic avenue for treating inflammatory diseases.

From this study, we identified several critical issues that need further focus. Firstly, while we showed different functions between WT and CIRP^−/−^ exosomes of LPS-injected mice, it still remains inconclusive whether the differences are due to the direct effects of exosomal eCIRP or indirect exosomal changes caused by the presence or absence of CIRP. For example, the lack of CIRP might have also caused differences in other exosomal contents, which synergistically enhancing functional differences. Secondly, we have performed only two experimental models, endotoxemia and sepsis. Since eCIRP is involved in various diseases caused by different stimuli, it would be important to prove eCIRP can also be released via exosomes in other inflammatory conditions. Since increased serum levels of eCIRP are directly associated with the sepsis severity in patients (Zhou et al., 2015; Gurien et al., 2020), the clinical relevance of this study would further be supported by confirming the elevation of exosomal eCIRP in the samples of patients with inflammatory diseases.

In conclusion, eCIRP is released via exosomes and contributes to inflammation by inducing cytokine production and neutrophil migration. Therefore, targeting exosomal eCIRP has the potential to become a novel therapeutic avenue to treat inflammatory diseases.

## Data Availability

The original contributions presented in the study are included in the article. Further inquiries can be directed to the corresponding authors.

## Abbreviations

CLP, cecal ligation and puncture; DAMPs, damage-associated molecular patterns; eCIRP, extracellular cold-inducible RNA-binding protein; HMGB1, high mobility group box 1; LPS, lipopolysaccharides; PAMPs, pathogen-associated molecular patterns; TLR, Toll-like receptor; TREM-1, triggering receptor expressed on myeloid cells-1.
